# *Ex Vivo* Innate Immune Cytokine Signature of Enhanced Risk of Relapsing Brucellosis

**DOI:** 10.1371/journal.pntd.0002424

**Published:** 2013-09-05

**Authors:** Kristyn E. Feldman, Paul M. Loriaux, Mayuko Saito, Iskra Tuero, Homarh Villaverde, Tenaya Siva, Eduardo Gotuzzo, Robert H. Gilman, Alexander Hoffmann, Joseph M. Vinetz

**Affiliations:** 1 Signaling Systems Laboratory, University of California San Diego, La Jolla, California, United States of America; 2 Division of Infectious Diseases, Department of Medicine, University of California San Diego School of Medicine, La Jolla, California, United States of America; 3 Alexander von Humboldt Institute of Tropical Medicine, Universidad Peruana Cayetano Heredia, Lima, Perú; 4 Departmento de Enfermedades Infecciosas y Tropicales, Hospital Nacional Cayetano Heredia, Lima, Perú; 5 Laboratorio de Investigación y Desarrollo, Facultad de Ciencias y Filosofía, Universidad Peruana Cayetano Heredia, Lima, Perú; 6 Department of International Health, Bloomberg School of Public Health, Johns Hopkins University, Baltimore, Maryland, United States of America; University of Tennessee, United States of America

## Abstract

**Background:**

Brucellosis, a zoonotic infection caused by one of the Gram-negative intracellular bacteria of the *Brucella* genus, is an ongoing public health problem in Perú. While most patients who receive standard antibiotic treatment recover, 5–40% suffer a brucellosis relapse. In this study, we examined the *ex vivo* immune cytokine profiles of recovered patients with a history of acute and relapsing brucellosis.

**Methodology/Principal Findings:**

Blood was taken from healthy control donors, patients with a history of acute brucellosis, or patients with a history of relapsing brucellosis. Peripheral blood mononuclear cells were isolated and remained in culture without stimulation or were stimulated with a panel of toll-like receptor agonists or heat-killed *Brucella melitensis* (HKBM) isolates. Innate immune cytokine gene expression and protein secretion were measured by quantitative real-time polymerase chain reaction and a multiplex bead-based immunoassay, respectively.

Acute and relapse patients demonstrated consistently elevated cytokine gene expression and secretion levels compared to controls. Notably, these include: basal and stimulus-induced expression of GM-CSF, TNF-α, and IFN-γ in response to LPS and HKBM; basal secretion of IL-6, IL-8, and TNF-α; and HKBM or Rev1-induced secretion of IL-1β, IL-2, GM-CSF, IFN-Υ, and TNF-α. Although acute and relapse patients were largely indistinguishable by their cytokine gene expression profiles, we identified a robust cytokine secretion signature that accurately discriminates acute from relapse patients. This signature consists of basal IL-6 secretion, IL-1β, IL-2, and TNF-α secretion in response to LPS and HKBM, and IFN-γ secretion in response to HKBM.

**Conclusions/Significance:**

This work demonstrates that informative cytokine variations in brucellosis patients can be detected using an *ex vivo* assay system and used to identify patients with differing infection histories. Targeted diagnosis of this signature may allow for better follow-up care of brucellosis patients through improved identification of patients at risk for relapse.

## Author Summary

Brucellosis is a disease caused by transmission of bacteria of the *Brucella* genus from infected animals to humans. The main route of infection occurs through consumption of contaminated dairy products or contact with infected animals. While most patients treated with antibiotics will be cured of the infection, between 5–40% of patients experience a relapse of brucellosis. The mechanisms underlying these recurring infections remain poorly understood. In this study, we examined blood cells from control donors, patients who previously had acute infections, and patients who previously had relapsing infections. We identified an inflammatory cytokine signature from measurements of unstimulated and stimulated cells that showed statistically significant differences between relapsing and non-relapsing brucellosis patients. Future applications of this assay system may allow for better follow-up care of brucellosis through the diagnosis of this cytokine signature and predictive or improved identification of patients at risk for relapse.

## Introduction

Brucellosis in humans is a zoonotic infection caused by Gram-negative facultative intracellular bacteria of the *Brucella* genus. Four species are typically responsible for human infections, *B. abortus*, *B. melitensis*, *B. suis*, *and B. canis*, and are transmitted from animal reservoirs including infected cows, goats or sheep, pigs, and dogs, respectively. Infection occurs by ingestion of contaminated unpasteurized milk or cheese or through contact with blood or materials from infected animals [1]. *B. melitensis* is recognized as not only the most virulent species, needing only a few organisms (10–100) to establish infection, but also the predominant species responsible for the brucellosis burden in Perú [2], [3]. *Brucella* spp. are of particular interest because they are easily aerosolized, which is underscored by the designation of brucellosis as the most common laboratory-acquired infection [4] and *Brucella* spp. as a category B agent on the Centers for Disease Control bioterrorism hazard list.

Approximately 5–40% of patients treated for brucellosis suffer a relapse, with the wide variation in risk historically being attributed to the duration and combination of antibiotic treatment [5]. However, few investigations have focused on the variation of the innate immune reaction to *Brucella* spp. and its impact on the rate of relapse. While studies have examined the association of genetic polymorphisms in cytokines and other immunity-related genes with brucellosis susceptibility [6], [7], less emphasis has been placed on the overall functional cytokine reaction of patients who demonstrate brucellosis susceptibility or relapse.

*Brucella* spp. are able to survive and replicate within macrophages, and effective control of brucellosis requires a potent Th1 response to activate cellular mediated immunity which is driven by the production of IFN-γ, IL-2, and TNF-α [8]–[12]. A Th2 response, driven by IL-4 and IL-10, is detrimental to combating brucellosis as it promotes humoral immunity and suppresses macrophage activation [13], [14].

In this study, we examined the *ex vivo* cytokine profiles of patients with a history of brucellosis in the absence of stimuli and after toll-like receptor (TLR) and heat-killed *Brucella melitensis* (HKBM) stimulation. This approach is unique because we assessed human cytokine expression and secretion in fully recovered patient blood cells to determine if there is a brucellosis cytokine signature present at baseline, that may underlie a person's response to *B. melitensis* infection. While previous studies employ animal models, cell lines, or look at post-treatment serum cytokine levels [15], we assessed the *ex vivo* immune reaction of primary cells from human patients. We found that several cytokines showed altered expression and secretion in both unstimulated and stimulated conditions. Patients with a history of acute or relapsing brucellosis can be accurately identified by a robust inflammatory cytokine signature, months and even years after successful treatment. This signature consists of increased secretion of TNF-α and IL-2 in response to HKBM and LPS, IL-1β in response to Rev1 and LPS, IFN-γ in response to HKBM, and basal IL-6. This work demonstrates that cytokine variations in brucellosis patients can be detected using an *ex vivo* assay system and can be used to distinguish between relapse and acute patients. Targeted diagnosis of this signature may allow for improved treatment of brucellosis by identifying patients at risk for relapse.

## Methods

### Ethics

The study was approved by the Human Research Protection Program of the University of California, San Diego, and the Comité de Ética of Universidad Peruana Cayetano Heredia (UPCH), Lima, Perú. All patients provided written informed consent prior to enrollment in the study.

### Patients and healthy volunteers

Sixteen patients with a previously confirmed history of acute brucellosis (6 males and 10 females; 44.8±12.5 years, “acute”) and 6 patients previously diagnosed with relapsing brucellosis (2 male and 5 females; 39±15.2 years, “relapse”) were enrolled in the study. Brucellosis was confirmed by serology, positive culture, or both methods (Supporting Table S1). At the time of sample collection all patients were 18 years of age or older, had completed treatment and were asymptomatic for brucellosis for 6 months or more, had a normal physical examination, and showed no signs or symptoms of other illness. 11 healthy volunteers with no history of brucellosis were also enrolled as negative controls (5 males and 6 females; 30.8±7.3 years, “control”).

Volunteers provided 120 mL of venous blood or underwent leukapheresis. Peripheral blood mononuclear cells (PBMCs) were isolated using Ficoll Paque (GE Healthcare) as previously described [16].

### *Ex vivo* cell culture

Isolated PBMCs were cultured in RPMI-1640 (Sigma) with 10% fetal bovine serum at a density of 2.5×10^6^ cells per well of a 24-well plate at 37°C with 5% CO_2_. After isolation, cells were allowed to rest for 4 hours and were then stimulated with either PBS (resting, basal), a TLR4 agonist, lipopolysaccharide B5:055 from *Escherichia coli* (LPS, 1 µg/ml, Sigma), a TLR2/1 agonist, the synthetic triacylated lipoprotein Pam3CSK4 (1 µg/ml), a TLR3 agonist, low molecular weight polyinosine-polycytidylic acid (Poly(I∶C), 10 µg/ml), a TLR7/8 agonist, the imidazoquinoline compound R848 (3 µg/ml), a TLR9 agonist, the synthetic CpG ODN 1668 (CpG, 5 mM), heat-killed *Brucella melitensis* vaccine strain Rev1 (Rev1, 65 CFU/ml) or a heat-killed, virulent *B. melitensis* patient isolate (HKBM, 65 CFU/ml). All manipulations of live *Brucella melitensis* vaccine strain Rev1 and the *B. melitensis* patient isolate were carried out under BSL3 conditions at UPCH, Lima, Peru. After 18 h of stimulation, the supernatant was removed and preserved at −80°C and the cells were washed with PBS and frozen for subsequent RNA isolation.

### RNA extraction and cDNA synthesis

After the culture supernatant was removed, PBMCs were washed in PBS, centrifuged, and the cell pellets were frozen at −80°C. Cells were thawed, lysed, homogenized, and total RNA was extracted using the QIAshredder and RNeasy kits per the manufacturer's instructions (Qiagen). RNA was eluted in 30 µl of RNase-free water, and 1 µg was reverse-transcribed into cDNA using the iScript cDNA synthesis kit according to the manufacturer's instruction (Bio-Rad).

### Quantitative real-time PCR and gene expression

Quantitative real-time PCR (qPCR) was performed to measure the mRNA expression level of the housekeeping gene GAPDH, and several inflammatory cytokines (GM-CSF, IFN-γ, IL-1β, IL-10 and TNF-α). Using a CFX384 Real-Time Detection System (Bio-Rad), each reaction was performed in triplicate in a final reaction volume of 5 µl, including 2.5 µl SsoAdvanced SYBR Green Supermix (Bio-Rad), 1.0 µl cDNA template, and 1.0 µl (100 nM final concentration) of each primer. Primers were designed for each gene using Primer3 (Supporting Table S2). After amplification, threshold cycle (C_T_) values were generated using the Bio-Rad CFX Manager Software 1.6. The fold change of gene expression was calculated as previously described [17].

### Cytokine protein secretion levels

A multiplex bead-based immunoassay was used to quantify cytokine levels secreted into the culture supernatant after stimulation. Using the Human Cytokine 10-Plex Panel for the Luminex platform, the following cytokines were measured according to the manufacturer's instruction: GM-CSF, IFN-γ, IL-1β, IL-2, IL-4, IL-5, IL-6, IL-8, IL-10 and TNF-α (Invitrogen). Briefly, either recombinant protein standards or 50 µl of each culture supernatant sample were first incubated, in duplicate, with antibody-conjugated fluorophore beads, and then with protein-specific biotinylated antibodies. Finally, following the addition of Streptavidin-RPE, samples were analyzed using the Bio-Plex 200 system (Bio-Rad). Data analysis was performed using the manufacturer provided software and the included recombinant proteins were used to generate standard curves to determine the sensitivity of the assay.

### Statistical methods

Significance values were calculated using the R software environment for statistical computing. For each pairwise comparison, Welch's t-test was used to estimate the probability that the two samples have equal mean. Probabilities less than 0.05 suggest significant differences between the two samples and are indicated by an asterisk.

### Classification and model selection

Prior to classification, all response variables were log_10_ transformed, centered, and scaled to unit variance. Unless otherwise stated, variables for which more than four patients were missing, or for which two or more patients belonging to the same category were missing, were discarded. Missing values in the remaining 70 response variables were imputed from their conditional means [18]. Specifically, for each missing value, a linear regression model was identified by forward model selection using Akaike's information criterion (AIC). Regressors were chosen from the 32 response variables for which no data was missing, including patient category. Forward selection was terminated when there was no further reduction in the AIC, or when the complexity of the model reached 12 regressors. Imputation by conditional means was chosen because of the relatively high correlation observed between variables [19], [20].

Linear discriminant analysis (LDA) was performed in R using the ‘lda’ function. Accuracy of the resulting linear discriminant function, or classifier, was then assessed using the ‘predict’ function in conjunction with leave-one-out cross-validation. To identify the optimal classifier for a given cross-section of the data, LDA was performed using all pairwise combinations of variables contained in the cross-section. Top-performing pairs, defined as those pairs of variables that trained a classifier with the highest accuracy, were then used to seed model selection. During model selection, a variable was chosen at each step whose inclusion in the classifier resulted in the greatest increase in accuracy, up to a backtracking factor of 0.03 (1 patient). Since a multiplicity of models could satisfy this selection criteria, each selection was performed 20 times. The model ultimately identified by forward selection was taken to be that which yielded the highest classification accuracy while using the fewest number of variables.

## Results

### Inflammatory cytokine gene expression is increased in brucellosis relapse patients

**Figure 1:**
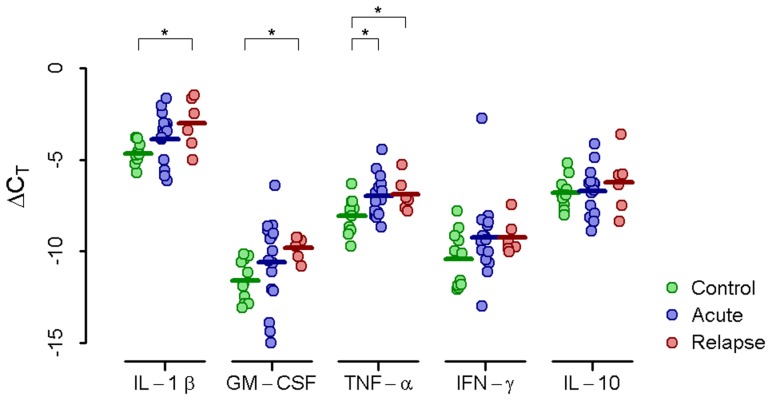
Basal PBMC cytokine gene expression. Relative basal amounts of IL-1β, GM-CSF, TNF-α, IFN-γ, and IL-10 mRNA compared to the housekeeping gene GAPDH (ΔC_T_) in unstimulated PBMCs from control donors or acute or relapse brucellosis patients (asterisk indicates *p*≤0.05).

To quantify the induction of cytokine gene expression in response to inflammatory stimuli, we first measured the resting, or basal, expression in unstimulated PBMCs. We found that basal expression of IL-1β and GM-CSF was significantly higher in relapse patients than in controls, while TNF-α was significantly higher in both acute and relapse patients compared to control (Figure 1).

**Figure 2:**
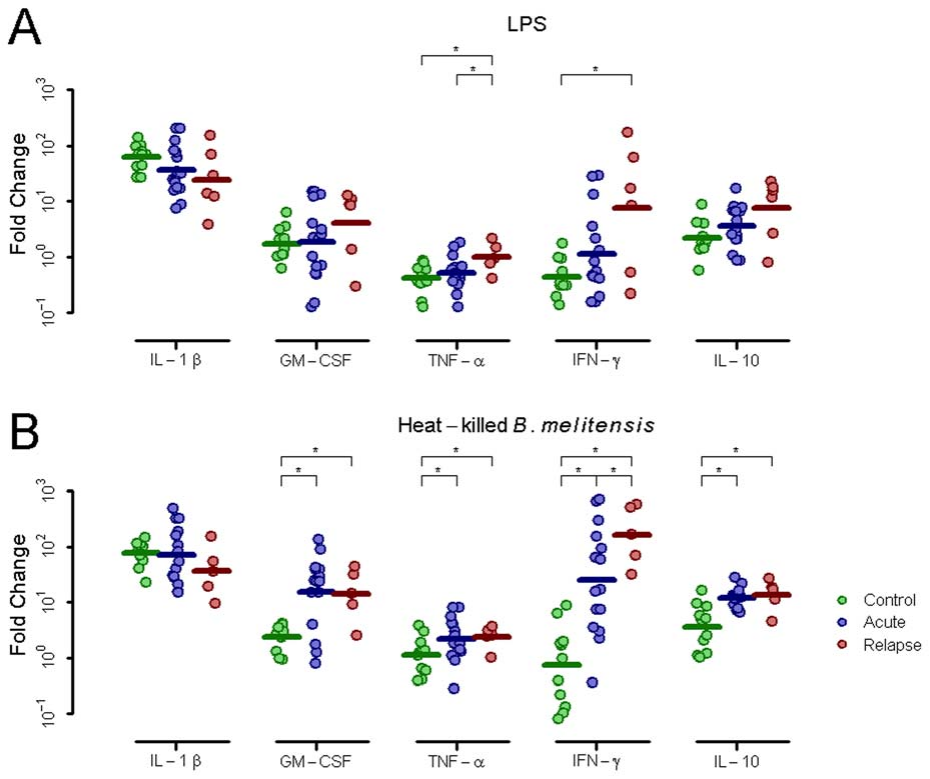
PBMC cytokine gene expression after stimulation. Fold change of gene expression for IL-1β, GM-CSF, TNF-α, IFN-γ and IL-10 in PBMCs from control donors or acute or relapse brucellosis patients after stimulation with (A) LPS (B) Heat-killed *B. melitensis* or (C) R848 (asterisk indicates *p*≤0.05).

Next, PBMCs were stimulated overnight with LPS, heat-killed *B. melitensis* (HKBM) or R848. In response to LPS, relapse patients exhibited higher expression of GM-CSF and IL-10 and significantly higher TNF-α and IFN-γ than either controls or acute patients (Figure 2A). This trend was also observed in response to HKBM, except relapse and acute patients exhibited similarly and significantly elevated levels of GM-CSF, TNF-α, and IL-10 (Figure 2B). Thus while cytokine gene expression in response to LPS appears to discriminate well between relapse and either acute or controls, the response to HKBM appears to discriminate between control subjects and either acute or relapse patients.

In summary, relapse patients uniquely demonstrated elevated basal IL-1β and GM-CSF expression compared to control donors. In comparison to both acute and control donors, relapse patients exhibit increased IFN-γ expression after HKBM stimulation and increased TNF-α expression after LPS.

### Inflammatory cytokine secretion is elevated in brucellosis relapse patients

**Figure 3:**
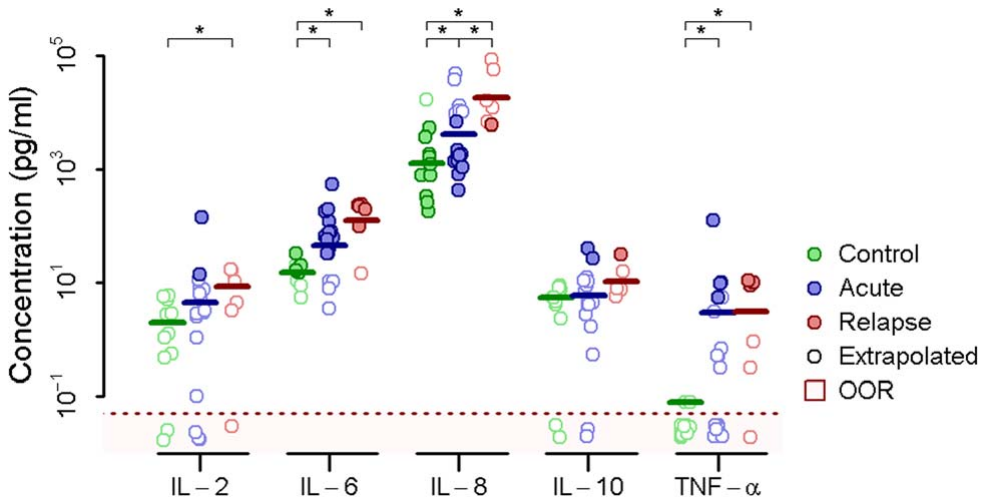
Basal PBMC cytokine secretion measured by multiplex immunoassay. IL-2, IL-6, IL-8, IL-10, and TNF-α secretion in unstimulated PBMCs from control donors or acute or relapse brucellosis patients (asterisk indicates *p*≤0.05). Concentrations indicated by open circles were extrapolated beyond the assay standard curve and values in the red shaded zone fell outside the observable range (OOR).

To test whether the differences observed in cytokine gene expression were also manifest in the synthesis and secretion of cytokine proteins, we used a multiplex bead-based immunoassay to quantify *ex vivo* cytokine secretion in the culture supernatant of unstimulated and stimulated PBMCs. We measured the concentrations of GM-CSF, IFN-γ, IL-1β, IL-2, IL-4, IL-5, IL-6, IL-8, IL-10, and TNF-α. In unstimulated cells we found that the basal secretion of IL-6, IL-8, and TNF-α was elevated in both acute and relapse patients compared to control subjects. IL-8 was higher in relapse patients than in acute patients, while basal IL-2 was increased in relapse patients compared to controls (Figure 3). All differences were significant (p<0.05).

**Figure 4:**
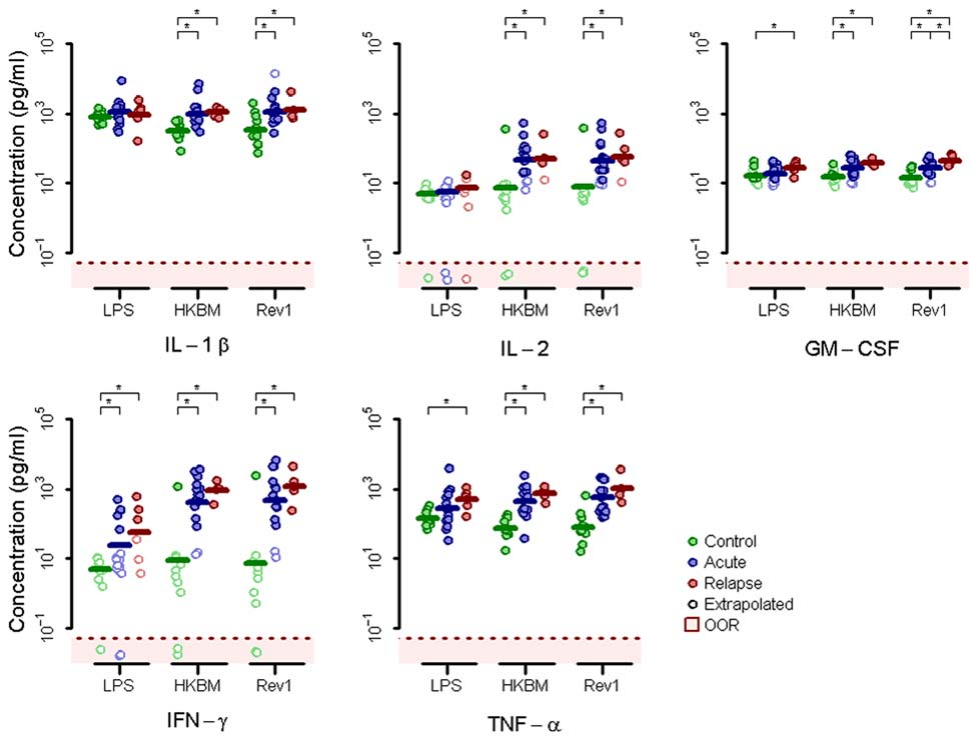
PBMC cytokine secretion after stimulation measured by multiplex immunoassay. IL-1β, IL-2, GM-CSF, IFN-γ, and TNF-α protein secretion by from control donors or acute or relapse brucellosis patients after stimulation with LPS, heat-killed *B. melitensis* (HKBM), or the heat-killed *B. melitensis* vaccine Rev1, as measured by multiplex immunoassay (asterisk indicates *p*≤0.05). Concentrations indicated by open circles were extrapolated beyond the assay standard curve and values in the red shaded zone fell outside the observable range (OOR).

Next we stimulated PMBCs with LPS, heat-killed *B. melitensis* (HKBM) or heat-killed *B. melitensis* vaccine strain Rev1 (Rev1). As observed in our gene expression data, after stimulating with HKBM, Rev1, or LPS, secretion of GM-CSF, IFN-γ, and TNF-α was significantly elevated in relapse patients compared to control subjects (Figure 4). Additionally, IL-1β and IL-2 secretion was significantly elevated in acute and relapse patients compared to control donors after both HKBM and Rev1, but not LPS, stimulation. Several of the cytokine concentrations measured in response to other stimuli fell out of the observable range of the assay (Supporting Figure S1).

### A robust cytokine signature accurately distinguishes relapsing from non-relapsing patients and controls

To test whether the differences observed in cytokine gene expression and protein secretion were sufficient to accurately discriminate between patients that did and did not experience a relapse in brucellosis, we trained a linear discriminant classifier using different cross-sections of the data and assessed its accuracy by leave-one-out cross validation. Linear discriminant analysis (LDA) is a supervised learning method that maximizes separation in the data – defined here as the ratio of variances between patient categories to the variance within – using a linear recombination of response variables, in this case our observed gene expression or cytokine secretion measurements. Using LDA in conjunction with a model selection strategy allowed us to ask whether a subset of the response variables that we assayed could accurately classify patients as control, acute, or relapse.

First, cross-sections of the cytokine gene expression and protein secretion data were chosen such that all response variables were of the same cytokine or generated using the same stimulus. We refer to these as “cytokine” and “stimulus” cross-sections, respectively. A classifier trained on a cytokine cross-section is said to be trained “across stimuli”, and *vice versa*. Response variables for which more than four patients were missing, or for which two or more patients belonging to the same category were missing, were discarded. Missing values in the remaining 70 response variables were imputed from their conditional means [18]. Linear discriminant functions were then identified for each cross-section using a forward model selection strategy with backtracking (see Methods).

On average, we found that higher classification accuracy was achieved by training across stimuli than across cytokines. Training across the four gene expression or eight protein secretion stimuli yielded accuracies of 0.679±0.073 and 0.642±0.119, respectively, compared to 0.598±0.045 and 0.606±0.116 across cytokines (Figures S2, S3, S4). This result is likely due to the higher cross-correlation observed between cytokines in response to a single stimulus, compared to the cross-correlation observed in a single cytokine in response to multiple stimuli.

Second, we observed that the cytokine secretion assay was superior at discriminating between acute and relapse patients compared to gene expression. With expression, only the IFN-γ cross-section correctly classified more than one relapse patient (Figure S2D). Conversely, four cytokine secretion cross-sections (IL-1, IL-6, IL-10, and TNF-α) and two stimulus cross-sections (Pam3CSK4 and R848) correctly classified half or more relapse patients (Figures S3, S4). This result is likely due to better separation in the response variables between acute and relapse patients in the cytokine secretion data compared to gene expression (Figure S5).

**Figure 5:**
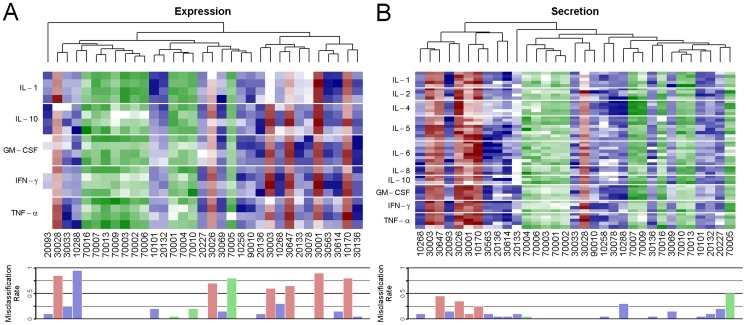
Hierarchical clustering of patients by gene expression or cytokine secretion. Control (green), acute (blue), and relapse (red) patients were clustered hierarchically by Euclidean distance in their scaled gene expression (A) or cytokine secretion (B) profiles (see Methods). Response variables are grouped by cytokine, indicated in the left margin, and values are indicated by luminosity. Misclassification rates for each patient after 20 model selection runs are indicated underneath the corresponding patient code (see Supporting Table S1).

Indeed, clustering the patients hierarchically by Euclidean distance in their gene expression or cytokine secretion profiles, we found that the gene expression profile for every relapse patient most closely matches that of an acute patient (Figure 5A). Similarly, control subject 70005 and acute patient 10288 cross-cluster with acute and control subjects, respectively. Consequently, these seven patients are misclassified in over half of the 20 qPCR models identified by forward selection. In contrast, five of the six relapse patients cluster together according to their cytokine secretion profile, resulting in significantly better classification performance (Figure 5B). Among the other patients, control subject 70005 and acute patient 10288 were again the most often misclassified, suggesting that these two may be outliers in their respective patient categories.

**Figure 6:**
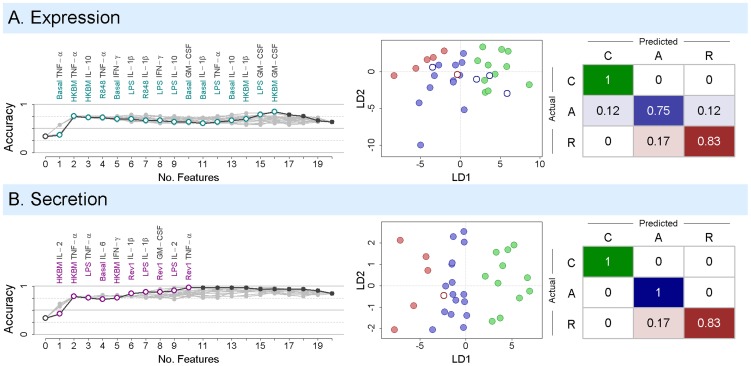
Model selection and classification results. Response variables were selected and a linear discriminant classifier was trained using the transformed gene expression (A) or cytokine secretion data (B). The left panels show the results of 20 model selection runs, with the best-performing classifier highlighted in color. The variable included after each step in the forward selection is listed for the optimal model. Zero features is equivalent to random guessing. The center column shows all 33 patients after being mapped by the first (LD1) and second (LD2) linear discriminant functions used by the best-performing classifier. Classification performance is summarized by the confusion matrix on the right. This matrix gives the proportion of (C)ontrol, (A)cute, and (R)elapse patients that were correctly (on-diagonal) and incorrectly classified (off-diagonal).

Examining the optimal gene expression model identified by forward selection, we found that it classified 28 of 33 patients correctly. Distinguishing between acute and relapse patients was the primary source of misclassification, with 83% relapse sensitivity (one false negative), but 71% precision (two false positives) (Figure 6A). In contrast, four cytokine secretion models correctly classified 32 of 33 patients. These models also classified five of six relapse patients correctly, but with perfect precision and fewer variables than gene expression (Figure 6B). Interestingly, these models all share the following eight response variables: TNF-α and IL-2 in response to HKBM and LPS, IL-1β in response to Rev1 and LPS, IFN-γ in response to HKBM, and basal IL-6. Pairing these variables with, for example, IL-1β and GM-CSF in response to HKBM, or TNF-α and GM-CSF in response to Rev1, achieves 97% patient classification accuracy. We therefore propose that these variables constitute an innate immune cytokine signature for accurate identification of patients at risk for brucellosis relapse.

## Discussion

Here we present evidence that patients with a history of acute-and-cleared or relapsing brucellosis can be distinguished with a robust inflammatory cytokine signature even months or years after successful treatment. Currently, under standard treatment, many patients experience relapsing brucellosis, the cause of which remains poorly understood. In this study we stimulated PBMCs from patients with a past history of acute or relapsing brucellosis and measured *ex vivo* innate inflammatory cytokine expression and secretion to determine if at a clinically normal baseline there was a cytokine signature that might be associated with relapsing infection.

*Brucella* spp. are intracellular pathogens whose effective control and elimination requires a potent cell-mediated Th1 immune response [9], [21], [22]. We found that relapse brucellosis patients demonstrated higher basal IL-1β and GM-CSF gene expression compared to control donors, increased IFN-γ expression after heat-killed *B. melitensis* (HKBM) stimulation and higher TNF-α expression after LPS stimulation compared to both acute brucellosis patients and control donors. Surprisingly, this indicates relapse patients are capable of inducing the expression of cytokines needed to mount a Th1 response. However increased IL-10 gene expression after stimulation with HKBM in both acute and relapse brucellosis patients, but not after LPS stimulation, may suggest a possible *Brucella* spp. specific elevated Th2 response. Th2 cytokines like IL-10 have been shown to downregulate immunity to *Brucella* spp. [23], [24].

Additionally, relapse patients produced more TNF-α protein compared to control donors and secrete more GM-CSF compared to both groups. Indeed, previous studies indicate GM-CSF secretion can stimulate IL-1β and TNF-α secretion by monocytes after *in vitro B. abortus* challenge [25]. Taken together, the *ex vivo* innate immune cytokine expression and secretion of acute or relapse patients indicates a functional and Th1-dominated response. IL-2, TNF-α, and IFN-γ secretion have previously been shown to be increased during brucellosis [26], [27], and recent studies also suggest that adequate levels are required for control of the infection as genetic polymorphisms in these genes may increase susceptibility to, or duration of, disease [6], [28]. In accordance with our findings, others have shown elevated IFN-γ after *ex vivo B. melitensis* antigen stimulation in patients less than one year after diagnosis [29]. Here we confirm that this remains true even several years after the resolution of infection.

Though gene expression of the Th2 cytokine IL-10 was elevated in some brucellosis patients, IL-10 protein secretion was not significantly altered in these patients under any stimulation condition; IL-4, another important Th2 cytokine, was not highly secreted in any condition (Supporting Figure S1). However, one key limitation of the study was the multiplex approach used to determine cytokine protein levels: several of the cytokines measured in the assay fell above or below the standard range defined in the manufacturer's protocol and some concentration values were extrapolated or not detected. Due to the limited quantity of patient sample and culture supernatant, individual optimization for each cytokine and standard in the 10-cytokine kit was not possible. To address this issue in future studies, multiplex kits with improved standard ranges could be used or individual conventional ELISA assays might be useful for key cytokines which still fall outside the detection of the multiplex assay.

In summary, this study demonstrates that innate immune cytokine variations can be detected between patients with a history of acute or relapsing brucellosis and control donors using an *ex vivo* assay system. Standard clinical methods for monitoring brucellosis treatment outcomes remain unreliable: antibody titers used for serological diagnosis of brucellosis and circulating *B. melitensis* DNA load used for diagnosis by PCR, have been shown to persist for years after successful treatment [30]–[34]. In contrast, we show that an *ex vivo* cytokine signature can accurately distinguish between relapse and acute patients, and may provide a novel approach to monitor clinical outcomes. Further work would be required to validate this *ex vivo* assay as a method for predicting or confirming actively relapsing infections.

## Supporting Information

Figure S1 **PBMC cytokine secretion after stimulation measured by multiplex immunoassay.** Secretion of IL-1β, IL-2, IL-4, IL-6, IL-8, IL-10, GM-CSF, IFN-γ, and TNF-α in PBMCs from control donors or acute or relapse brucellosis patients without stimulation (basal) or after stimulation with Pam3CSK4, Poly(I∶C), LPS, R848, CpG, HKBM, or Rev1. Concentrations indicated by open circles were extrapolated beyond the assay standard curve and values in the red shaded zone fell outside the observable range (OOR).
(TIF)

Figure S2 **Classifiers identified by forward model selection using all cytokine (A–E) and stimulus (F–I) cross-sections in the gene expression data.** Each panel illustrates the model selection (left) and resulting classifier performance (right) for cytokines (A) GM-CSF (B) IL-10 (C) TNF- α (D) IFN- γ and (E) IL-10 and stimuli (F) Basal (no stimulus) (G) HKBM (H) LPS and (I) R848. For model selection, the accuracy of the resulting classifier is given as a function of the number of variables incorporated. The results of 20 selections are shown for each cross-section, with the best-performing classifier highlighted in bold. The identity of each variable incorporated into the best-performing classifier is indicated above its corresponding index. Zero features is equivalent to random guessing. For each cross-section, the confusion matrix generated by the best-performing classifier is shown at right. This matrix gives the proportion of the 11 (C)ontrol, 16 (A)cute, and 6 (R)elapse patients that were correctly and incorrectly classified.
(TIF)

Figure S3 **Classifiers identified by forward model selection for all cytokine cross-sections in the cytokine secretion data.** Each panel illustrates the model selection (left) and resulting classifier performance (right) for cytokines (A) IL-1β (B) IL-2 (C) IL-4 (D) IL-5 (E) IL-6 (F) IL-8 (G) IL-10 (H) GM-CSF (I) IFN- γ and (J) TNF- α. See Figure S2 for details.
(TIF)

Figure S4 **Classifiers identified by forward model selection for all stimulus cross-sections in the cytokine secretion data.** Each panel illustrates the model selection (left) and resulting classifier performance (right) for stimuli (A) Basal (no stimulus) (B) Pam3CSK4 (C) Poly(I∶C) (D) LPS (E) R848 (F) CpG (G) HKBM and (H) Rev1. See Figure S2 for details.
(TIF)

Figure S5 **Data separation in the gene expression and cytokine secretion data sets.** Scaled, log_10_-transformed response variables were taken from the full gene expression data set (left) and the partial, imputed cytokine secretion data set (right), where no variable was missing more than four values and no patient category was missing more than one value (see Methods). Variables for which relapse patients exhibited a mean value less than that of control subjects were inverted (multiplied by −1). The density of the resulting variables was estimated using the ‘density’ function in R, then overlayed by patient category: control (green), acute (blue) and relapse (red).
(TIF)

Table S1 **Clinical characteristics of patient population.**
(XLS)

Table S2 **Primers used for quantitative real-time PCR.**
(XLS)
