# Supplementary figures and images for: Ex Vivo Innate Immune Cytokine Signature of Enhanced Risk of Relapsing Brucellosis
Source: PLoS Negl Trop Dis. 2013 Sep 5;7(9):e2424. doi: 10.1371/journal.pntd.0002424 (PMC3764229; doi:10.1371/journal.pntd.0002424)

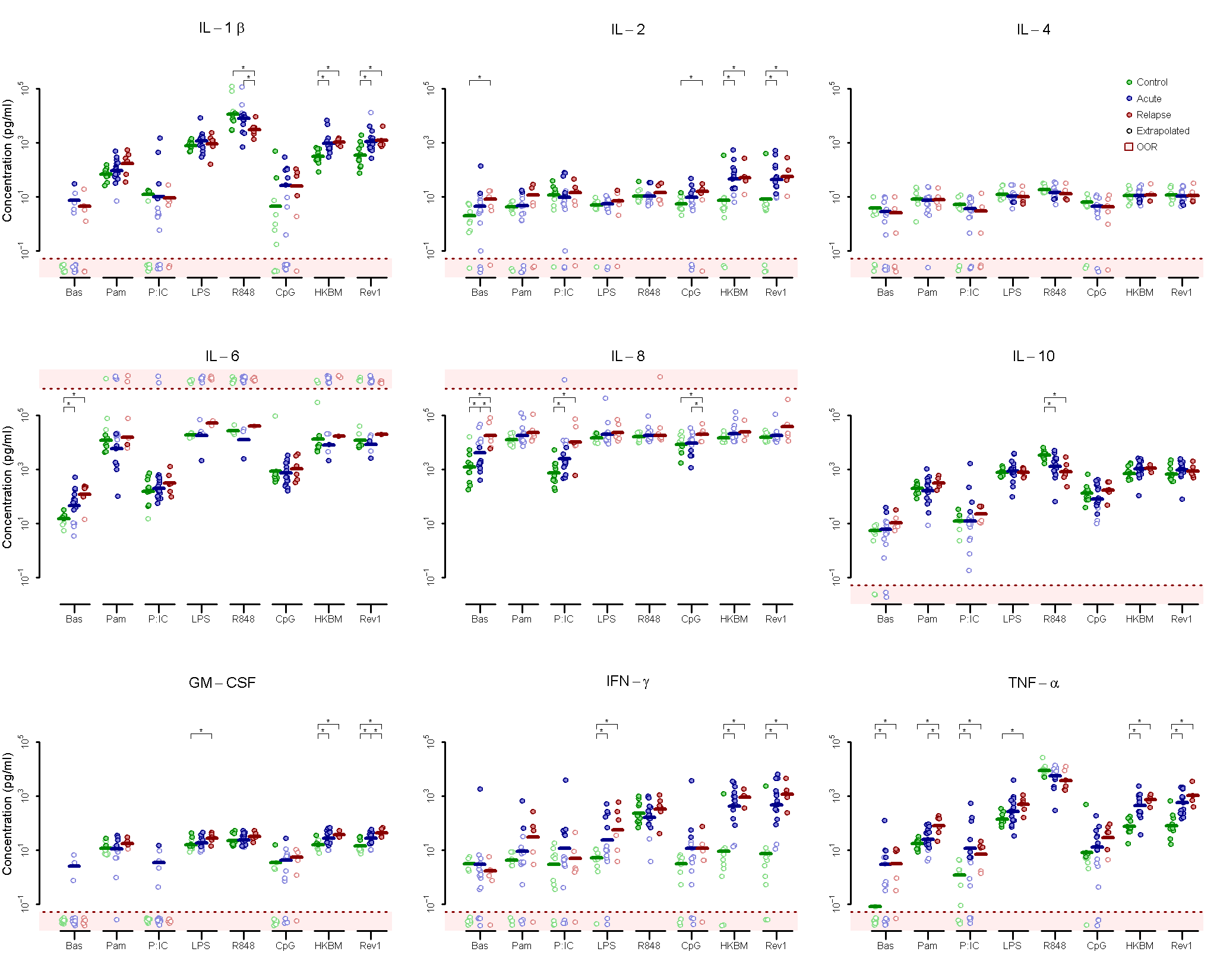

Supplement: Figure S1 — PBMC cytokine secretion after stimulation measured by multiplex immunoassay. Secretion of IL-1β, IL-2, IL-4, IL-6, IL-8, IL-10, GM-CSF, IFN-γ, and TNF-α in PBMCs from control donors or acute or relapse brucellosis patients without stimulation (basal) or after stimulation with Pam3CSK4, Poly(I∶C), LPS, R848, CpG, HKBM, or Rev1. Concentrations indicated by open circles were extrapolated beyond the assay standard curve and values in the red shaded zone fell outside the observable range (OOR). (TIF) [file pntd.0002424.s001.tif]

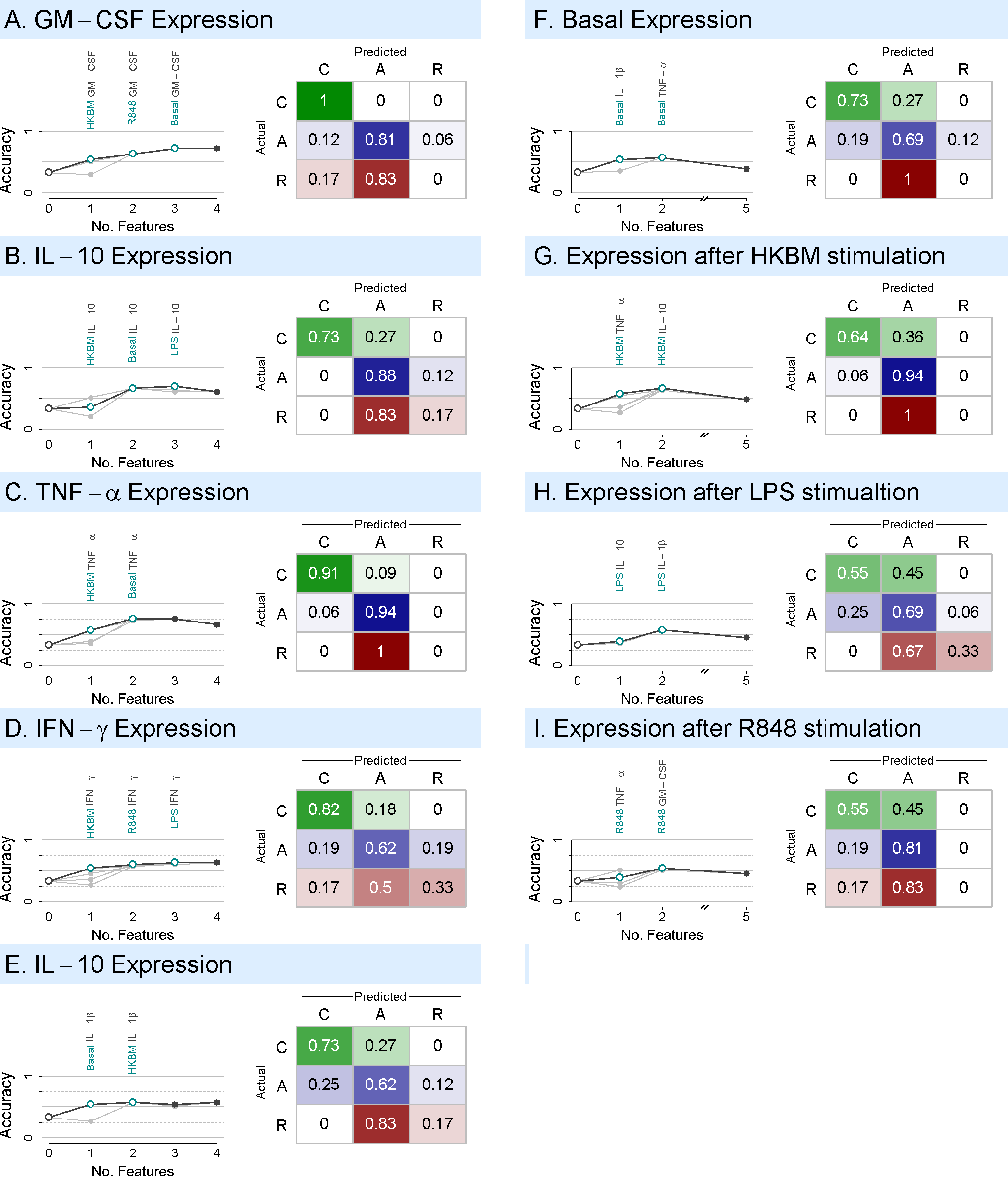

Supplement: Figure S2 — Classifiers identified by forward model selection using all cytokine (A–E) and stimulus (F–I) cross-sections in the gene expression data. Each panel illustrates the model selection (left) and resulting classifier performance (right) for cytokines (A) GM-CSF (B) IL-10 (C) TNF- α (D) IFN- γ and (E) IL-10 and stimuli (F) Basal (no stimulus) (G) HKBM (H) LPS and (I) R848. For model selection, the accuracy of the resulting classifier is given as a function of the number of variables incorporated. The results of 20 selections are shown for each cross-section, with the best-performing classifier highlighted in bold. The identity of each variable incorporated into the best-performing classifier is indicated above its corresponding index. Zero features is equivalent to random guessing. For each cross-section, the confusion matrix generated by the best-performing classifier is shown at right. This matrix gives the proportion of the 11 (C)ontrol, 16 (A)cute, and 6 (R)elapse patients that were correctly and incorrectly classified. (TIF) [file pntd.0002424.s002.tif]

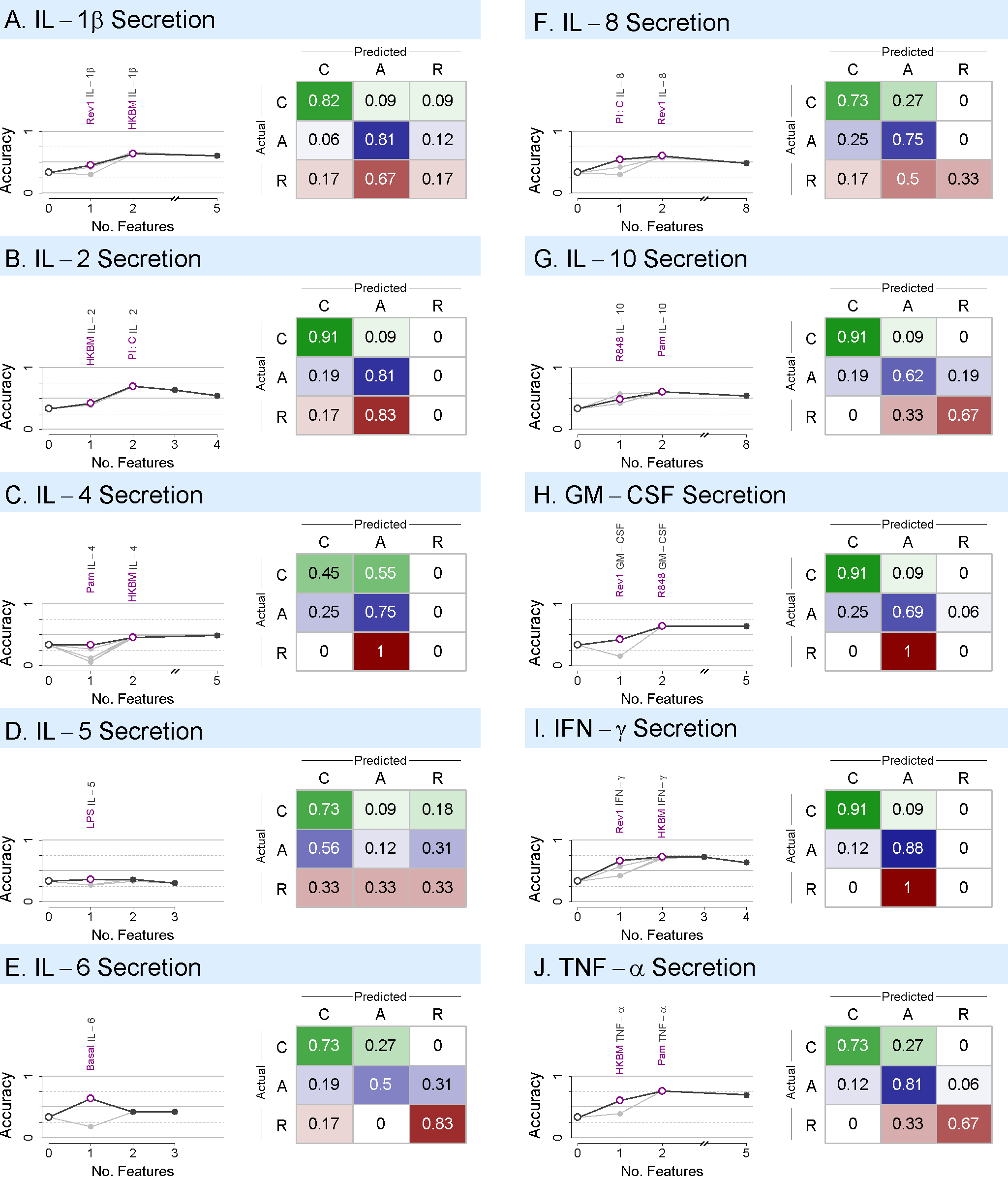

Supplement: Figure S3 — Classifiers identified by forward model selection for all cytokine cross-sections in the cytokine secretion data. Each panel illustrates the model selection (left) and resulting classifier performance (right) for cytokines (A) IL-1β (B) IL-2 (C) IL-4 (D) IL-5 (E) IL-6 (F) IL-8 (G) IL-10 (H) GM-CSF (I) IFN- γ and (J) TNF- α. See Figure S2 for details. (TIF) [file pntd.0002424.s003.tif]

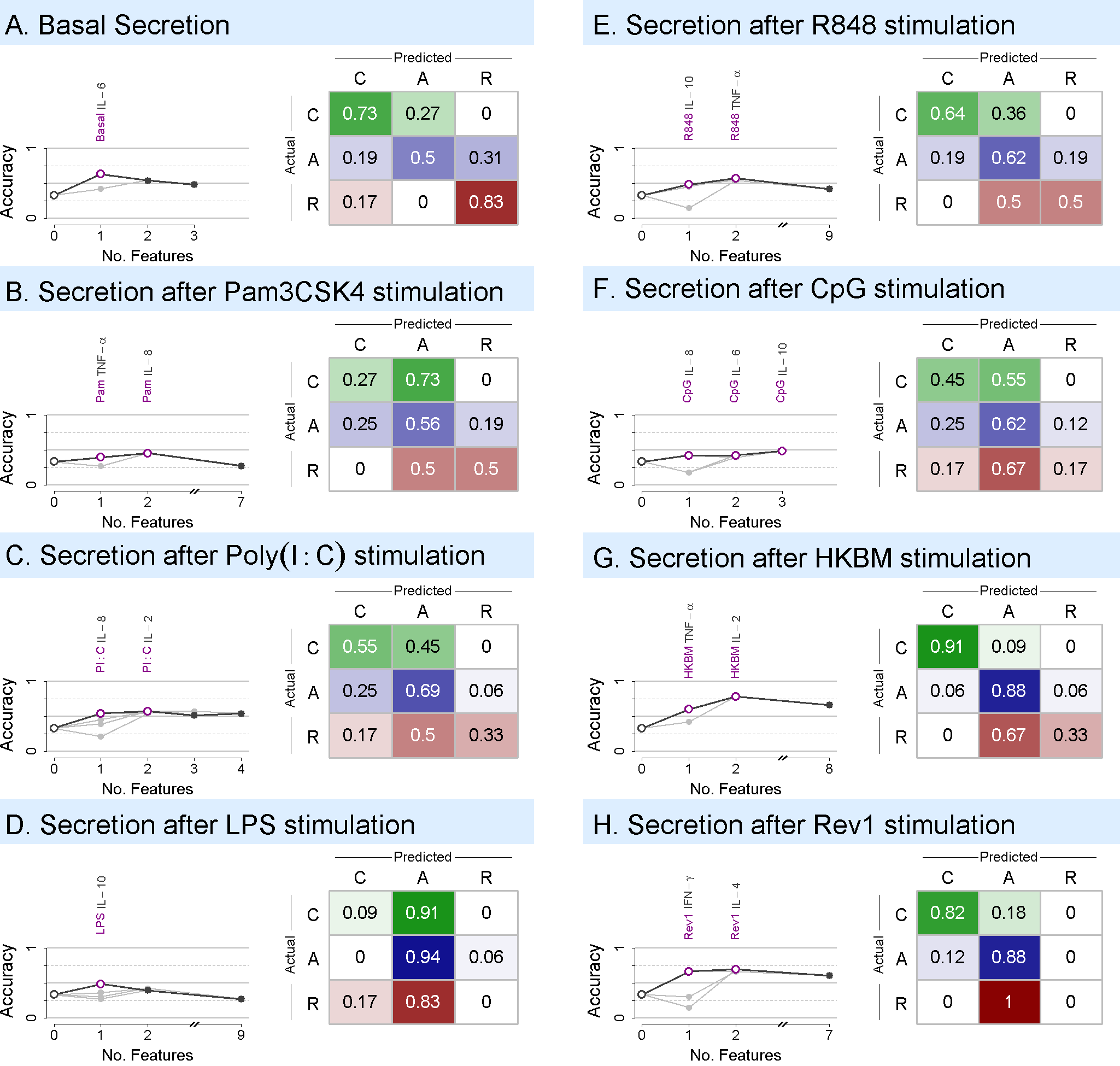

Supplement: Figure S4 — Classifiers identified by forward model selection for all stimulus cross-sections in the cytokine secretion data. Each panel illustrates the model selection (left) and resulting classifier performance (right) for stimuli (A) Basal (no stimulus) (B) Pam3CSK4 (C) Poly(I∶C) (D) LPS (E) R848 (F) CpG (G) HKBM and (H) Rev1. See Figure S2 for details. (TIF) [file pntd.0002424.s004.tif]

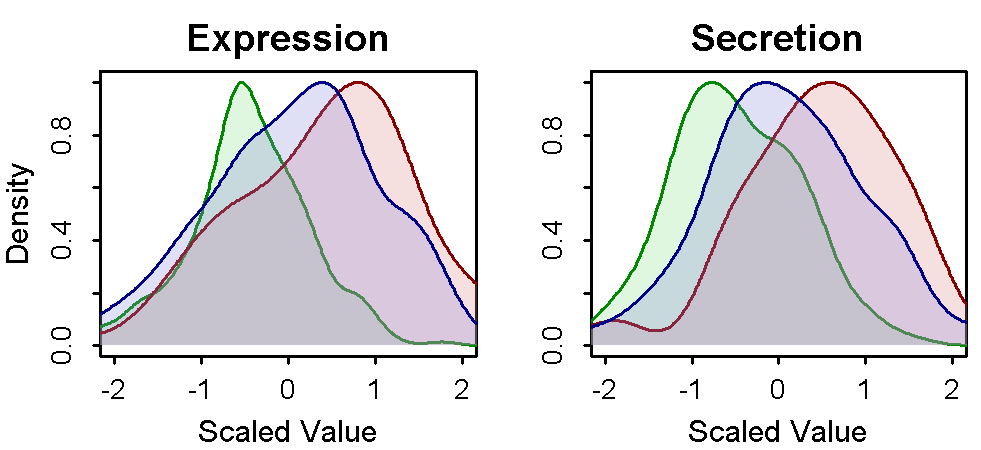

Supplement: Figure S5 — Data separation in the gene expression and cytokine secretion data sets. Scaled, log10-transformed response variables were taken from the full gene expression data set (left) and the partial, imputed cytokine secretion data set (right), where no variable was missing more than four values and no patient category was missing more than one value (see Methods). Variables for which relapse patients exhibited a mean value less than that of control subjects were inverted (multiplied by −1). The density of the resulting variables was estimated using the ‘density’ function in R, then overlayed by patient category: control (green), acute (blue) and relapse (red). (TIF) [file pntd.0002424.s005.tif]
